# Red Wine Grape Pomace Attenuates Atherosclerosis and Myocardial Damage and Increases Survival in Association with Improved Plasma Antioxidant Activity in a Murine Model of Lethal Ischemic Heart Disease

**DOI:** 10.3390/nu11092135

**Published:** 2019-09-06

**Authors:** Katherine Rivera, Francisca Salas-Pérez, Guadalupe Echeverría, Inés Urquiaga, Sara Dicenta, Druso Pérez, Paula de la Cerda, Leticia González, Marcelo E. Andia, Sergio Uribe, Cristián Tejos, Gonzalo Martínez, Dolores Busso, Pablo Irarrázaval, Attilio Rigotti

**Affiliations:** 1 Department of Nutrition, Diabetes, and Metabolism, School of Medicine, Pontificia Universidad Católica, Santiago 8330033, Chile (K.R.) (F.S.-P.) (G.E.) (D.B.); 2 Center of Molecular Nutrition and Chronic Diseases, School of Medicine, Pontificia Universidad Católica, Santiago 8330033, Chile (I.U.) (S.D.) (D.P.) (P.d.l.C.); 3 Biomedical Imaging Center, Department of Electrical Engineering, School of Engineering, Pontificia Universidad Católica, Santiago 7820436, Chile (L.G.) (M.E.A.) (S.U.) (C.T.) (P.I.); 4 Millennium Nucleus for Cardiovascular Magnetic Resonance, Santiago 7820436, Chile; 5 Department of Radiology, School of Medicine, Pontificia Universidad Católica, Santiago 8330033, Chile; 6 Department of Electrical Engineering, School of Engineering, Pontificia Universidad Católica, Santiago 7820436, Chile; 7 Division of Cardiovascular Diseases, School of Medicine, Pontificia Universidad Católica, Santiago 8330033, Chile

**Keywords:** atherosclerosis, oxidative stress, magnetic resonance imaging, fiber and antioxidants, grape pomace

## Abstract

A healthy dietary pattern and high quality nutrient intake reduce atherosclerotic cardiovascular disease risk. Red wine grape pomace (RWGP)—a rich natural source of dietary fiber and antioxidants—appears to be a potential functional food ingredient. The impact of a dietary supplementation with RWGP flour was evaluated in atherogenic diet-fed SR-B1 KO/ApoER61^h/h^ mice, a model of lethal ischemic heart disease. SR-B1 KO/ApoER61^h/h^ mice were fed with atherogenic (high fat, cholesterol, and cholic acid, HFC) diet supplemented with: (a) 20% chow (HFC-Control), (b) 20% RWGP flour (HFC-RWGP), or (c) 10% chow/10% oat fiber (HFC-Fiber); and survival time was evaluated. In addition, SR-B1 KO/ApoER61^h/h^ mice were fed for 7 or 14 days with HFC-Control or HFC-RWGP diets and plasma lipid levels, inflammation, oxidative damage, and antioxidant activity were measured. Atherosclerosis and myocardial damage were assessed by histology and magnetic resonance imaging, respectively. Supplementation with RWGP reduced premature death, changed TNF-α and IL-10 levels, and increased plasma antioxidant activity. Moreover, decreased atheromatous aortic and brachiocephalic plaque sizes and attenuated myocardial infarction and dysfunction were also observed. These results suggest that RWGP flour intake may be used as a non-pharmacological therapeutic approach, contributing to decreased progression of atherosclerosis, reduced coronary heart disease, and improved cardiovascular outcomes.

## 1. Introduction

Atherosclerosis is an inflammatory condition induced by the accumulation of cholesterol deposits within the arterial wall triggering an inflammatory response, leading to a pathological intimal lesion, which contributes to the development of ischemic cardiovascular disease (CVD) [1,2,3]. In primary and secondary prevention, statins are the lipid-lowering drug class of choice in order to reduce the risk of morbidity and mortality due to atherosclerotic CVD (ASCVD) [4]. Despite their effectiveness, more than half of patients who use statins still develop ischemic events due to coronary artery and cerebrovascular diseases [5].

Healthy lifestyle choices also have a significant beneficial effect on the initiation and progression of CVD. Among these, dietary patterns with a high consumption of fruit and vegetables—rich in dietary fiber and natural antioxidants—have shown benefits in prevention of ASCVD and other chronic diseases [6]. While soluble fiber reduces intestinal cholesterol absorption and, consequently blood cholesterol levels [7], natural antioxidants inactivate reactive oxygen species (ROS) and reduce oxidative damage, a process involved in the beginning and development of chronic diseases, including atherosclerosis [8,9,10].

Multiple experimental studies and clinical trials have used polyphenols and other antioxidant-enriched products as therapeutic agents. For example, consumption of pomegranate juice concentrate, a source of potent tannin and anthocyanin antioxidants, reduces oxidative damage markers in diabetic patients [11] and also lowers vascular oxidative stress and attenuates atherosclerosis in SR-B1 (scavenger receptor class b type 1)/ApoE (apolipoprotein E) double KO mice [12]. In addition, grapes contain a large variety of natural antioxidants, including resveratrol, catechin, epicatechin, and proanthocyanidins as well as fiber from seeds and skin [13]. Dietary interventions with grape skin and/or seed extracts have been used in models of atherosclerosis to evaluate their potential effect on this disease [13]. Health benefits of moderate wine consumption have also been studied in CVD, atherosclerosis, and hypertension, among others with some protective associations; however, no conclusive recommendations exist regarding its intake [14]. Moreover, daily alcohol drinking has been associated with increased mortality [15]. Studies conducted in ApoE-deficient mice supplemented with de-alcoholised wine have shown a reduction in adhesion molecules, pro-inflammatory cytokines, and atherosclerotic lesions [16], suggesting ethanol-independent effects.

Red wine grape pomace (RWGP) can be obtained from waste byproducts—composed by skin, stems, and seeds that are rich in phenolic compounds and dietary fiber—during the winemaking process. This byproduct can be dried and milled to produce a functional ingredient as RWGP flour. Daily consumption of RWGP-enriched foods has shown beneficial effects on antioxidant and metabolic parameters in animals and humans [17,18,19]. However, most studies only focus on blood lipid levels and oxidation analyses, rather than evaluating atherosclerosis progression and actual ischemic outcomes from coronary heart disease or cardiovascular death in appropriate animal models. In this context, the aim of this study was to evaluate the impact of dietary supplementation with RWGP flour on ASCVD and premature death and its potential protective mechanisms in atherogenic diet-fed SR-B1 KO/ApoER61^h/h^ mice, a diet-induced model of occlusive atherosclerosis, coronary heart disease, and cardiac death [20].

## 2. Materials and Methods

### 2.1. Animals

SR-B1 KO/ApoER61^h/h^ mice, originally obtained from Dr. Monty Krieger (Massachusetts Institute of Technology (MIT), Cambridge, MA, USA), were maintained in the animal facility of the School of Medicine at Pontificia Universidad Católica de Chile under controlled light, temperature, and humidity conditions, with free access to water and control chow diet (5% fat, 22% protein, 0.022% cholesterol, 4.0% fiber; Prolab RMH 3000; PMI Feeds Inc., Brentwood, CA, USA). Food intake and body weight were controlled daily throughout the study period. All procedures described were performed following recommendations from the Guide for the Care and Use of Laboratory Animals published by the US National Institutes of Health and were approved by the Ethics and Animal Welfare Committee from Pontificia Universidad Católica de Chile (protocols #14-036 and #170131001).

### 2.2. Experimental Diets

A high fat, high cholesterol, and cholic acid-containing (HFC) atherogenic diet (15.5% fat, 20.6% protein, 1.25% cholesterol, 0.5% cholic acid, 3.1% fiber; Cocoa Test Diet 57BB, St Louis, MO, USA) was used as a basis for preparation of experimental diets. Briefly, HFC diet was supplemented with: 20% chow (HFC-Control), 20% RWGP flour (HFC-RWGP), or 10% chow/10% oat fiber (HFC-Fiber), and the composition of each experimental diet was determined by proximate food analysis (Table 1). Chow diet and oat fiber were used as atherogenic diet dilution and fiber addition controls, respectively. RWGP, containing 47.7% dietary fiber, was previously obtained from Cabernet Sauvignon grapes [18] and stored at −20 °C until used. Oat fiber, containing 92.8% dietary fiber, was obtained commercially (Canadian Harvest^®^ Oat Fiber 780, SunOpta Ingredients Group, Bedford, MA, USA). The nutritional information of RWGP [18] and oat fiber is reported in Supplemental Table S1.

### 2.3. Survival Study

For the survival study, female and male SR-B1 KO/ApoER61^h/h^ mice at two months of age were randomly distributed into three intervention groups. Animals were fed with HFC-Control (*n* = 20), HFC-RWGP (*n* = 19), or HFC-Fiber (*n* = 21) diets and the survival rate was determined by tabulating death of mice as a function of time in Kaplan–Meier curves as previously described [21]. All animals were visually inspected twice a day to assess their health status. Criteria for euthanasia were based on guidelines considering poor general appearance and abnormal behavior (e.g., ruffled fur, abnormal gait, reduced food and water intake, decreased body weight, or reduced activity). Spontaneous death or humanitarian endpoint-euthanized animals were represented in the survival curves.

### 2.4. Prospective Detailed Assessment Study

For this study, we used adult SR-B1 KO/ApoER61^h/h^ mice at baseline (day 0) (*n* = 5) or after feeding HFC-Control or HFC-RWGP diets for 7 (*n* = 7) or 14 days (*n* = 7), respectively. For in vivo cardiovascular imaging, mice were anaesthetized using isoflurane (1.5%–2% for induction and 0.5%–1% for maintenance) and imaged in prone position before and 30 min after intravenous administration of 0.03 mmol/kg gadofosveset as a contrast agent (Ablavar, Lantheus Medical Imaging, North Billerica, MA, USA).

### 2.5. Tissue Sampling

For tissue and blood sampling, mice were euthanized with a mixture of ketamine:xylazine (150:10 mg/kg intraperitoneally (i.p.)) after 4-h fasting. Blood was obtained by abdominal vena cava puncture using heparin as anticoagulant. Plasma was prepared by low-speed centrifugation of blood and kept at −20°C. Hearts were inspected macroscopically and presence of at least 1 myocardial white patch was recorded as ‘infarcted’ whereas the absence of white patches was considered as ‘non-infarcted’. Aorta and hearts were embedded in optimal cutting temperature (OCT) solution (Sakura Finetek USA, Inc., Torrance, CA, USA), frozen in liquid nitrogen, and stored to −80 °C. Liver and spleen were also extracted and weighed in all animals.

### 2.6. Blood Lipoprotein Separation

Whole plasma was fractionated by fast protein liquid chromatography (FPLC) using a Superose-6 10/300 GL column (GE Healthcare Life Sciences, Pittsburgh, PA, USA) as previously described [22]. The first 15 mL were discarded and the next 40 fractions of 300 µL each, containing the eluted lipoproteins according to their particle size, were recovered. Samples were stored at −20 °C until use.

### 2.7. Blood Cholesterol Determinations

Plasma total and lipoprotein cholesterol levels were quantified through an enzymatic method described previously, with some modifications [23]. Briefly, 10 μL of plasma or 300 µL of FPLC fractions were incubated for 30 min at 37 °C with 1% Triton X-100 in 0.5 M Tris-HCl pH 7.6, 50 mM phenol, 50 mM 4-chlorophenol, 0.37% sodium cholate, 0.04% 4-aminoantipyrine, 0.35 U/mL cholesterol esterase, 1.1 U/mL peroxidase (all reagents from Sigma Aldrich, St. Louis, MO, USA), and 0.1 U/mL cholesterol oxidase (Calbiochem, La Jolla, CA, USA). The absorbance was measured at 500 nm in a plate reader and cholesterol concentrations were calculated using a standard curve.

### 2.8. Determination of Plasma Levels TNF-α and IL-10

Tumor necrosis factor alpha (TNF-α) and interleukin 10 (IL-10) levels were measured through immunodetection kits according to the manufacturer’s instructions (R&D Systems, Minneapolis, MN, USA). The data obtained for each sample were interpolated in a standard curve.

### 2.9. Liver Function Tests

Liver injury was estimated by measuring plasma activities of alanine transaminase (ALT) and gamma-glutamyltransferase (GGT) with a commercial kit through a UV method, according to the manufacturer’s instructions (HUMAN Diagnostics Worldwide, Wiesbaden, Denmark).

### 2.10. Analysis of High-Density Lipoprotein (HDL)-Containing Plasma Antioxidant Activity

Antioxidant activity was evaluated with a dihydrorhodamine (DHR)-based fluorescent assay in HDL-containing plasma after non HDL lipoprotein removal, as previously described with modifications [24,25]. Briefly, plasma samples were mixed with 20 μM dextran sulfate and 1 M MgCl_2_ to obtain final concentrations of 2 μM dextran sulfate and 50 mM MgCl_2_. After 10 min incubation and centrifugation at 8000 rpm at 4 °C, HDL-containing plasma was recovered, and total cholesterol concentration was measured. HDL-containing plasma volumes holding 10 µg of total cholesterol were incubated with 25 µL of 50 μM DHR solution in 96-well plates. The fluorescence emitted by the spontaneous oxidation of this reagent in the absence or presence of the plasma samples was measured with a fluorimeter at 2 min intervals during one hour at 37 °C (λEXC = 485 nm; λEM = 538 nm). The percentage of DHR oxidation was calculated from kinetic slopes of experimental group samples relative to control reactions without plasma addition.

### 2.11. Determination of Plasma Malondialdehyde Levels

Malondialdehyde (MDA) levels were analyzed as a marker of oxidative damage in plasma lipids and quantified as previously described with some modifications [26]. Plasma was deproteinized with 5% trichloroacetic acid (TCA) and supernatant was treated with fresh 0.6% thiobarbituric acid (TBA) and incubated for 45 min at 90 °C. The mixture was cooled down at room temperature and injected in a reverse-phase high pressure chromatograph (HPLC) (Merck-Hitachi 7000 series, Merck-Hitachi, Darmstadt, Germany). The separation was made using an Inertsil ODS-3 column (GL Sciences, Tokyo, Japan) and a mobile phase (65% sodium phosphate buffer pH 7 and 35% methanol). UV–Vis photodiode array detector and a fluorescence detector (λEXC = 515 nm; λEM = 550  nm) were used for measurements. MDA concentrations were calculated by interpolating the area of the peak corresponding to the sample adduct MDA-TBA.

### 2.12. Histological Characterization of Aortic Root Atherosclerosis

Aortic roots obtained from hearts of treated mice were cut into serial 10-μm cross sections and stained for neutral lipids with Oil Red O (Sigma-Aldrich, St. Louis, MO, USA) as follows. Frozen sections were fixed with neutral formalin for 10 min, then placed in 60% isopropanol for 1 min, and stained with 60% Oil Red O solution for 30 min. Then, sections were washed with water and 60% isopropanol, rinsed, and counterstained with Meyer’s hematoxylin (Sigma-Aldrich, St. Louis, MO, USA). Stained sections were dehydrated and mounted with 10% gelatin. Images were captured using an optical microscope (Eclipse E200, Nikon Instruments, Inc., Melville, NY, USA). Lesion sizes were estimated using the Image J software in several sections across aortic valves (3–4 serial sections).

### 2.13. Cardiovascular Magnetic Resonance Imaging

In vivo cardiovascular magnetic resonance imaging (MRI) was performed using a 1T ICON MR scanner (Bruker, Billerica, MA, USA) at baseline (day 0) as well as 7 and 14 days after diet consumption. Images were acquired for visualization of aortic arch as well as brachiocephalic and carotid arteries, and for evaluation of cardiac function. Following a 3D gradient echo scout scan, a black-blood RARE sequence was run in the aortic arch and supra-aortic vessels (Supplemental Figure S4). An inversion recovery image—pre and post contrast—was acquired to visualize arterial vessel contrast enhancement: echo time (TE)/repetition time (TR): 2.6 ms/6.4 ms; flip angle: 25°; field of view (FOV): 30 × 30 × 8 mm; matrix acquisition: 256 × 256; in-plane resolution: 0.12 × 0.12 mm; inversion time: 400 ms, time between subsequent inversion pulses: 1000 ms). Electrocardiographic (ECG)-gated T1-weighted cine FLASH images for cardiac function analysis were acquired to study contractile cardiac function (TE/TR: 3 ms/19.8 ms; flip angle: 25°; FOV: 25 × 25 × 5 mm; matrix acquisition: 116 × 116; in-plane resolution: 0.21 × 0.21 mm).

### 2.14. MRI Data Analysis

The volume of contrast agent uptake in the aorta and brachiocephalic arteries (BCA) was calculated from inversion recovery MRI images by manually segmenting the visually enhanced region of the vessel wall (OsiriX Foundation, Geneva, Switzerland). Cardiac function was assessed from Cine-FLASH images calculating left end-systolic and end-diastolic ventricle volumes and the ejection fraction through OsiriX software (Pixmeo SARL, Geneva, Switzerland).

### 2.15. Statistical Analyses

The survival rate was determined by tabulating the death of mice as a function of time with Kaplan–Meier curves. The log-rank (Mantel–Cox) test was used for comparison among three groups. For additional analyses, normality of data was determined with the Kolmogorov–Smirnov test. Results are shown as mean ± standard deviation (SD) or median with interquartile range (IQR) (Q1 and Q3) as specified, where *n* indicates the sample size. The statistical significance of differences between proportions was evaluated with the Chi-squared test. Comparisons between two groups at baseline and day 7 and day 14 post-dietary feeding were performed using two-way ANOVA for nonparametric data with Sidak post hoc test for multiple comparisons. When the comparisons were made at the end of the intervention (day 14), analysis was performed using the Mann–Whitney U test for unpaired nonparametric data. Differences were considered significant when * *p* < 0.05 between supplemented diets, ^#^ *p* < 0.05 compared to day 0 (baseline); and ^&^ *p* < 0.05 compared to day 7. GraphPad Prism 8.0.1 (GraphPad Software, La Jolla, CA, USA) was used for statistical data analysis.

## 3. Results

### 3.1. RWGP Supplementation Attenuated Premature Death in Atherogenic Diet-Fed SR-B1 KO/ApoER61^h/h^ Mice

**Figure 1:**
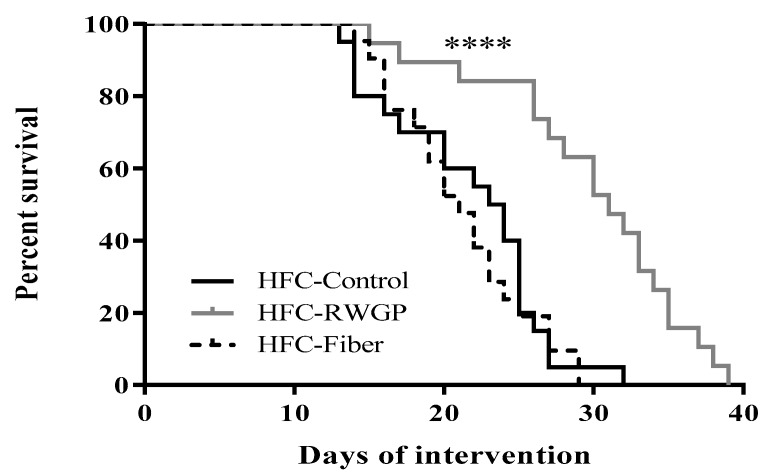
Effect of RWGP supplementation on survival of SR-B1 KO/ApoER61^h/h^ mice fed atherogenic diet. Kaplan–Meier survival curves of HFC-Control (*n* = 20), HFC-RWGP (*n* = 19), and HFC-Fiber (*n* = 21) groups obtained from three independent experiments. **** indicates *p* < 0.0001 between HFC-RWGP vs. HFC-Control and HFC-Fiber groups based on log-rank test.

The survival study was performed to investigate the effect of RWGP supplementation on lifespan of atherogenic diet-fed SR-B1 KO/ApoER61^h/h^ mice. The atherogenic diet caused early death in SR-B1 KO/ApoER61^h/h^ mice as previously described [20,27]. However, RWGP addition showed a significant improvement on animal lifespan with a median survival of 31 days (solid gray line) in comparison with HFC-Control (23.5 days; solid black line) or HFC-Fiber groups (21 days; dashed line) (*p* < 0.0001) (Figure 1). Interestingly, HFC-Fiber group did not improve lifespan. This finding indicates that the effect of RWGP was not related to mere dilution of the atherogenic diet, mere presence of fiber, or changes in overall food/water intake among groups (data not shown), but it was most likely due to specific components of the RWGP flour [18] and HFC-RWGP diet (Table 1).

### 3.2. Total and Lipoprotein Cholesterol Levels were Unaffected by RWGP in Atherogenic Diet-Fed SR-B1 KO/*ApoE*R61^h/h^ Mice

**Figure 2:**
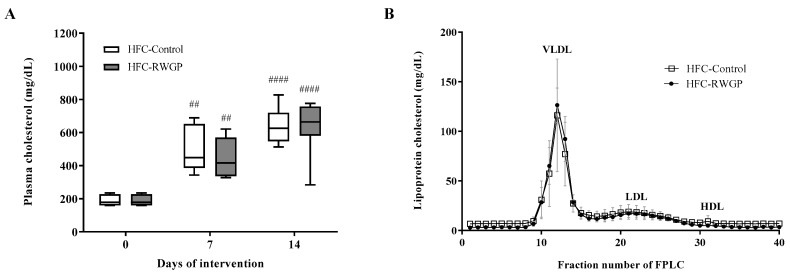
Effect of RWGP supplementation on plasma total and lipoprotein cholesterol levels of SR-B1 KO/ApoER61^h/h^ mice fed with atherogenic diet. (**A**) Data for total plasma cholesterol are shown in box plots (box, 25th to 75th percentiles; whiskers, median and interquartile range (IQR)) (*n* = 5–7). ^##^ *p* < 0.01 and ^####^ *p* < 0.0001 compared to day 0 based on two-way ANOVA. (**B**) Distribution of cholesterol content in plasma lipoproteins. Data represent averages and error (*n* = 5–7).

Body weights did not differ between groups during the dietary interventions (Supplemental Figure S1). HFC-Control diet-fed SR-B1 KO/ApoER61^h/h^ mice presented hypercholesterolemia after 7 and 14 days of diet consumption compared to baseline in accordance with previous observations from our group [28,29] (Figure 2A). Similar increases in total cholesterol levels were found for HFC-RWGP diet-fed mice. Cholesterol determinations in different plasma lipoprotein fractions separated by gel filtration did not show differences between both groups at day 7 (data not shown) or at day 14 (Figure 2B).

### 3.3. RWGP Supplementation Modulated Inflammatory Cytokines in Atherogenic Diet-Fed SR-B1 KO/*ApoE*R61^h/h^ Mice

**Figure 3:**
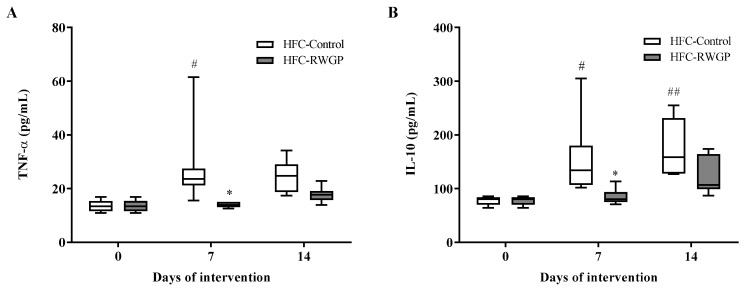
Impact of RWGP supplementation on plasma inflammation markers of SR-B1 KO/ApoER61^h/h^ mice fed with atherogenic diet. (**A**) TNF-α and (**B**) IL-10 levels at baseline and after 7 and 14 days of feeding HFC-supplemented diet. Data are shown in box plots (box, 25th to 75th percentiles; whiskers, median and IQR) (*n* = 5–7). ^#^ *p* < 0.01 and ^##^ *p* < 0.01 compared to day 0; * *p* < 0.01 vs. HFC-Control based on two-way ANOVA.

HFC-Control-fed mice showed a significant increase in TNF-α levels after 7 days of intervention compared to day 0 (*p* < 0.05), which was prevented with RWGP supplementation at day 7 (*p* < 0.05) (Figure 3A). On the other hand, HFC-Control diet also increased IL-10 levels after 7 (*p* < 0.05) and 14 (*p* < 0.01) days of consumption and RWGP supplementation resulted in a significant decrease (by 59.9%) in IL-10 levels at day 7, compared to HFC-Control diet (*p* < 0.05) (Figure 3B). After 14 days of RWGP supplementation, TNF-α and IL-10 showed a non-significant trend to reduce levels compared to the HFC-Control diet.

In addition, the pro-inflammatory state exhibited by SR-B1 KO/ApoER61^h/h^ animals fed with an atherogenic diet correlated with enlarged spleens and the addition of RWGP attenuated these changes after 7 or 14 days of diet consumption compared to the HFC-Control group (Supplemental Figure S2A). Previous observations have also suggested the key role of the liver in the inflammatory response evoked by dietary cholesterol [30]. However, SR-B1 KO/ApoER61^h/h^ animals fed with HFC-RWGP diet did not show changes in liver weights after 7 or 14 days, compared to HFC-Control diet (Supplemental Figure S2B). We also did not observe differences on ALT levels, a liver damage marker enzyme, between HFC-Control or HFC-RWGP groups at different time points (Supplemental Figure S3A). Interestingly, HFC-Control group showed high levels of GGT, another liver disease marker enzyme, after 14 days of diet intake (*p* < 0.05, compared to day 0), while no reversal of these alterations was observed in the HFC-RWGP group (Supplemental Figure S3B).

### 3.4. RWGP Supplementation Increases HDL-Containing Plasma Antioxidant Activity in Atherogenic Diet-Fed SR-B1 KO/*ApoE*R61^h/h^ Mice

**Figure 4:**
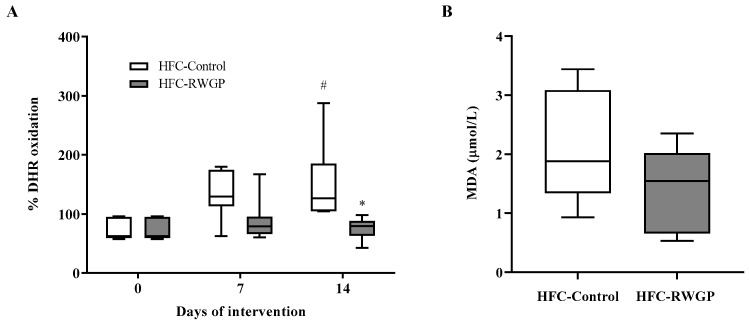
Effect of RWGP supplementation on high-density lipoprotein (HDL)-containing plasma antioxidant activity malondialdehyde (MDA) levels of SR-B1 KO/ApoER61^h/h^ mice fed with atherogenic diet. (**A**) % DHR oxidation at baseline and after 7 and 14 days of HFC diet. (**B**) Plasma MDA levels after 14 days of HFC diet intake. Data are shown in box plots (box, 25th to 75th percentiles; whiskers, median and IQR) (*n* = 5–7). ^#^ *p* < 0.05 compared to day 0; * *p* < 0.05 vs. HFC-Control based on two-way ANOVA.

We evaluated the effect of the RWGP supplementation on antioxidant activity from plasma containing HDL particles, but depleted of non-HDL lipoproteins, at different times. This parameter was analyzed using a cell-free assay that measures the ability of a sample to prevent spontaneous oxidation of DHR to fluorescent rhodamine. Antioxidant activity in plasma was significantly decreased in HFC-Control-fed animals after 14 days of dietary intervention, reflecting an increase of 102.6% in DHR oxidation (*p* < 0.05, compared to day 0). Interestingly, this decrease in plasma antioxidant activity induced by the atherogenic diet was fully prevented by dietary RWGP supplementation for 14 days as shown by a significant decrease to baseline levels of DHR oxidation in HFC-RWGP compared to HFC-Control diet (*p* < 0.05) (Figure 4A).

The presence of increased antioxidant activity in plasma at day 14 suggested that RWGP supplementation might have modulated lipid peroxidation. Thus, we evaluated plasma levels of MDA, a well-characterized oxidation product of polyunsaturated fatty acids present in proatherogenic oxidized low-density lipoproteins (LDL) [31]. The exposure to the HFC-RWGP diet for 14 days did not significantly change plasma MDA levels compared to HFC-Control group (Figure 4B).

### 3.5. Decreased Atherosclerotic Lesions and Improved MRI-Assessed Endothelial Function Induced by RWGP Supplementation in Atherogenic Diet-Fed SR-B1 KO/*ApoE*R61^h/h^ Mice

**Figure 5:**
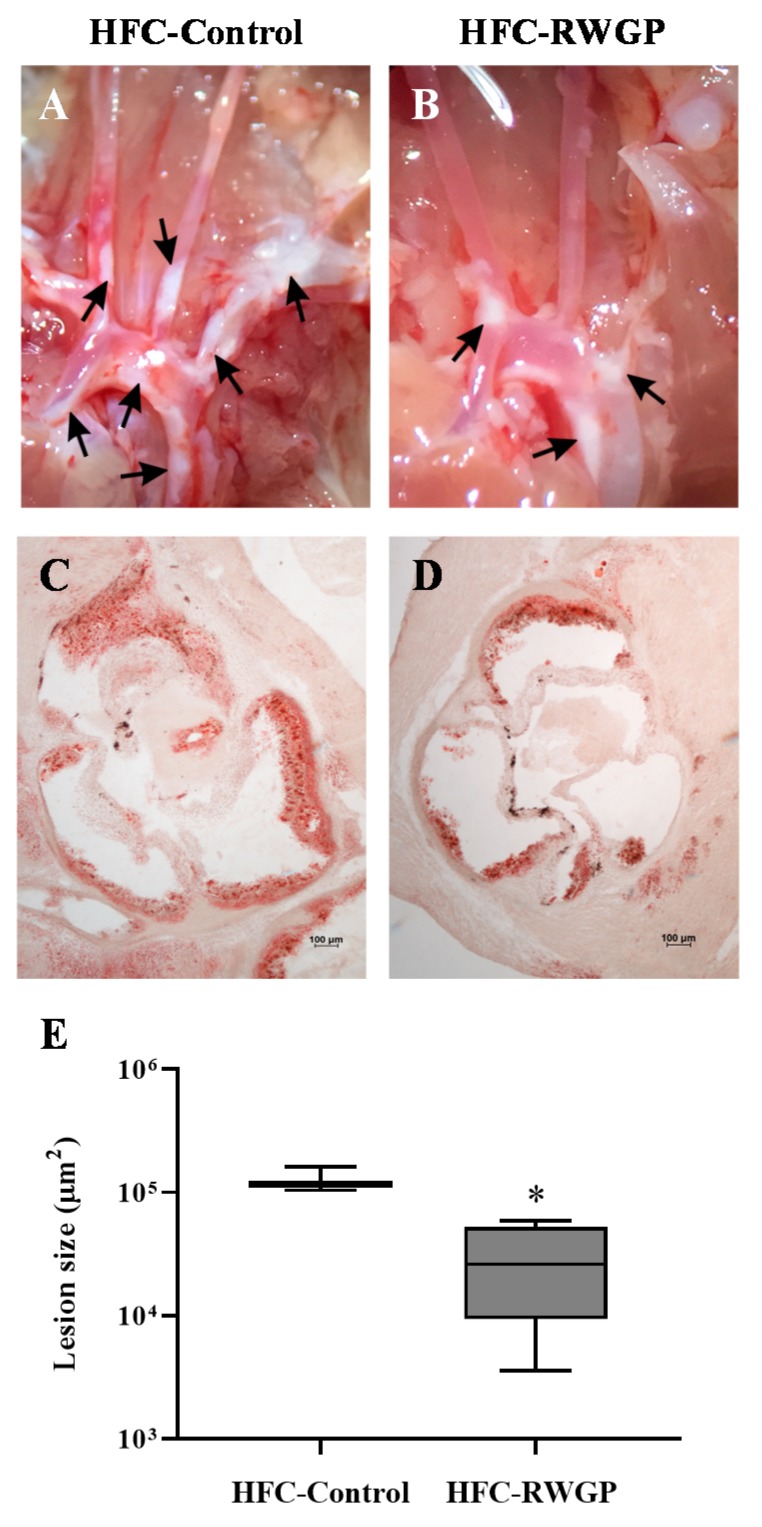
Effect of RWGP supplementation in atherosclerotic lesions of SR-B1 KO/ApoER61^h/h^ mice fed with atherogenic diet. Representative images obtained during dissections of aortic arches from mice fed HFC-Control (**A**) or HFC-RWGP (**B**) diets for 14 days. Representative cross sections of aortic roots stained with Oil Red O from HFC-Control- (**C**) or HFC-RWGP- (**D**) fed mice at day 14. (**E**) Quantitative analysis of aortic valve atherosclerotic lesion areas. Data are shown in box plots (box, 25th to 75th percentiles; whiskers, median and IQR) (*n* = 5–7). * *p* < 0.05 vs. HFC-Control based on two-way ANOVA.

Atheromatous lesions, evidenced as white patches in tissues during dissection of aortic arches, and histological sections of aortic roots stained with Oil Red O from HFC-Control and HFC-RGWP-fed mice at day 14 are shown in Figure 5.

SR-B1 KO/ApoER61^h/h^ mice fed with HFC-Control diet for 14 days exhibited formation of atherosclerotic plaques in several regions of the aortic arch and its branches (black arrows; Figure 5A). Interestingly, macroscopic inspection in the HFC-RGWP group evidenced a reduction in lesions compared to aortas from HFC-Control-fed mice (Figure 5B). As previously described [20], analyses of Oil Red O-stained histological cross sections of aortic roots from HFC-Control fed SR-B1 KO/ApoER61^h/h^ mice exhibited multiple, large atherosclerotic lesions (Figure 5C). Remarkably, Oil Red O stained areas in sections from HFC-RWGP mice were smaller than those observed in the control group (Figure 5D). Quantitative analysis of the cross-sectional area of aortic root atherosclerosis demonstrated a significant decrease in mean lesion area in HFC-RWGP mice at day 14, compared to HFC-Control group (*p* < 0.05) (Figure 5E).

**Figure 6:**
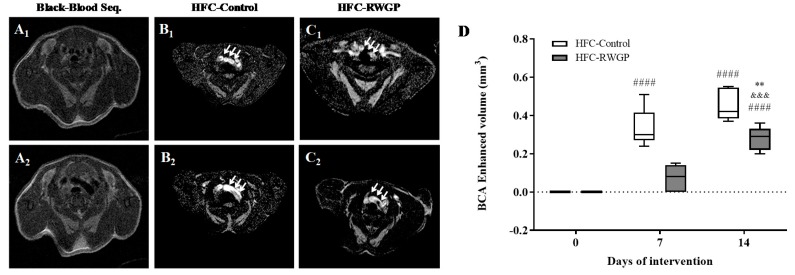
Effect of RWGP supplementation on vessel wall uptake of gadofosveset in SR-B1 KO/ApoER61^h/h^ mice fed with atherogenic diet. Representative black-blood axial magnetic resonance imaging (MRI) of the supra-aortic vessels (**A1**) and the aortic arch (**A2**). Inversion recovery MR images showing gadofosveset accumulation in vessel walls of mice fed HFC-Control (**B1**,**B2**) or HFC-RWGP (**C1**,**C2**) diets for 14 days. White arrows show areas of contrast uptake in aortic arch and supra-aortic vessels. (**D**) Quantification of contrast uptake in vessel walls at 14 days of dietary manipulations. Data are shown in box plots (box, 25th to 75th percentiles; whiskers, median and IQR) (*n* = 5–7). ^####^ *p* < 0.0001 compared to day 0; ^&&&^ *p* < 0.001 compared to day 7; ** *p* < 0.01 vs. HFC-Control based on two-way ANOVA.

On the other hand, animals at baseline (day 0) as well as 7 and 14 days after dietary interventions underwent noninvasive MRI scanning (Figure 6), which offered high spatial resolution for evaluation of vessel wall structure and functionality. A novel method for image contrast and acquisition was used in order to detect and follow the progression of endothelial damage in this mouse model of ASCVD. Gadofosveset is a clinically-approved gadolinium-based contrast agent that reversibly binds to serum albumin with prolonged retention within circulation. Under normal conditions, gadofosveset remains predominantly within the vascular lumen, but it may enter the vessel wall through dysfunctional endothelium and leaky neovessels [32] (Figure 6A–C).

Aortic arch and supra-aortic vessels of mice reconstructed from bright-blood MRI acquisition are shown in Supplemental Figure S4. Gadofosveset uptake in the aortic arch and the supra-aortic vessel wall—measured as BCA enhanced volume—significantly increased at day 7 and 14 in mice fed with HFC-Control diet (*p* < 0.0001, compared to day 0 for each time point), while contrast uptake was significantly decreased in RWGP-supplemented group at day 14, compared to HFC-Control during follow-up (*p* < 0.01) (Figure 6D).

### 3.6. RWGP Supplementation Attenuated Myocardial Dysfunction and Reduced Myocardial Infarction in Atherogenic Diet-Fed SR-B1 KO/*ApoE*R61^h/h^ Mice

**Figure 7:**
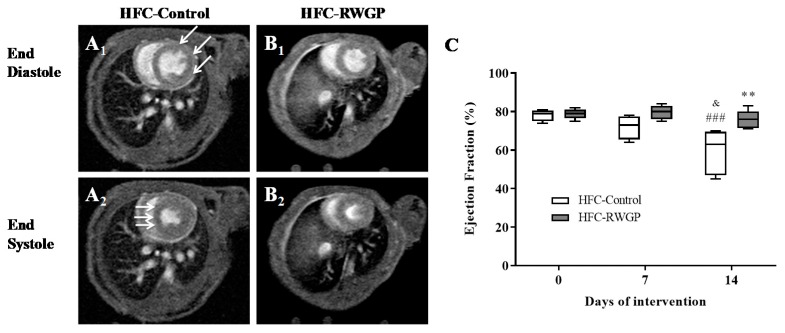
Impact of RWGP supplementation on contractile cardiac function of SR-B1 KO/ApoER61^h/h^ mice fed with atherogenic diet. Cardiac images were obtained by Cine-FLASH MR. Representative diastolic or systolic end pictures are shown for mice fed with HFC-Control (**A1**,**A2**) or HFC-RWGP diet (**B1**,**B2**) at day 14, respectively. White arrows indicate diastolic dysfunction (A1) and septum hypokinesia (A2) in HFC-Control mice. (**C**) Quantification of ejection fraction at baseline and after 7 and 14 days of dietary interventions. Data are shown in box plots (box, 25th to 75th percentiles; whiskers, median and IQR) (*n* = 5–7). ^###^ *p* < 0.001 compared to day 0; ^&^ *p* < 0.001 compared to day 7; ** *p* < 0.01 vs. HFC-Control based on two-way ANOVA.

Atherosclerotic lesions in coronary arteries are the pathological basis for ischemic complications leading to myocardial infarction. Cardiac function—measured as ejection fraction in the left ventricle—showed a progressive deterioration of contractile heart function in HFC-Control-fed mice (Figure 7A), being significantly decreased after 14 days of diet intake (*p* < 0.001) (Figure 7C). Mice from the HFC-RWGP group showed a significant restoration of systolic heart function at day 14 (Figure 7B) to normal levels compared with HFC-Control (*p* < 0.01) (Figure 7C).

These differences in heart function were consistent with the detection of infarcted myocardium in all of HFC-Control mice (100%) at 14 days of diet intake (Table 2). By contrast, only 1 out of 7 HFC-RWGP mice (14.3%) exhibited myocardial infarctions (*p* < 0.0001) (Table 2). These results indicated that animals fed with HFC-RWGP for 14 days reduced their risk of macroscopic ischemic heart disease by 86%.

## 4. Discussion

Although current pharmacological therapies to treat human ASCVD have successfully reduced morbidity and mortality, a remaining, and significant, residual risk supports the need for new research and therapeutic efforts [5,33]. The use of antioxidants has been envisioned as an additional strategy to treat this condition due to the contribution of oxidation-based mechanisms during CVD diseases [8,9,10,34]. 

Red wine polyphenols seem to have antioxidant, anti-inflammatory, blood pressure lowering effects [35] with potential applications as co-treatment to decrease residual risk in ASCVD. Nevertheless, human clinical trials have shown that these effects are not always achievable within the context of moderate alcohol consumption, due to low blood and tissue concentrations of polyphenolic metabolites reached after wine intake in the human body [36]. Additionally, standard biosynthetic antioxidant supplementation has not proven beneficial effects in clinical trials [37]. This latter suggests that new formulations, such as utilization of natural plant-based antioxidant fiber, may provide new opportunities for preclinical research and clinical development.

In the present study, we evaluated the impact of RWGP consumption in a mouse model that recapitulates the initiation, progression, and lethality of ASCVD [20]. Our results show that dietary RWGP supplementation increases the lifespan of SR-B1 KO/ApoER61^h/h^ mice fed with an atherogenic diet. We also established that changes in systemic inflammation and restoration of HDL-containing plasma antioxidant activity by RWGP consumption were associated with a significant reduction in atherosclerotic lesions and ischemic heart disease.

Our results indicated that only the HFC-RWGP, but not the HFC-fiber only, diet had an effect on survival, our primary study outcome, in a model of lethal coronary heart disease. RWGP used in this study contains a significant proportion of dietary fiber as well as functional antioxidants [18]. Identification and measurements of phenolic acid, flavonoid, and anthocyanin contents in RWGP extracts were previously reported [18]. Polyphenols (α-tocopherol, gallic acid (GA), catechin, flavonols, and malvidin) are the main antioxidants present in RWGP [18]. Similar findings were previously published with a combined supplementation of vitamin C and E, both well-known antioxidants, in atherogenic diet-fed SR-B1 KO/ApoER61^h/h^ mice [29]. Recent studies have revealed that GA has beneficial effects against several cardiovascular diseases, specifically GA, which suppresses cardiac hypertrophic remodeling and heart failure [38]. On the other hand, catechin [39] and flavonols [40]—obtained from green tea or red wine—decreased plasma oxidized LDL levels in apolipoprotein E-deficient mice and hypercholesterolemic mice. Also, malvidin has shown antioxidant and anti-inflammatory effects in vitro [41]. In addition to a direct protective effect of bioavailable antioxidants, RWGP may have also improved HDL antioxidant function and protein composition. Indeed, using this functional ingredient—as a supplement or incorporated into hamburgers—determined increased HDL antioxidant capacity and protein content in our own human studies (manuscript under preparation). Taken together, this evidence suggests that RWGP antioxidant components themselves or their effects on protein-dependent HDL function are possible mechanisms underlying the beneficial effects observed in this experimental model of severe atherosclerosis.

We also investigated some additional potential protective mechanism(s) of RWGP. In Wistar rats fed with a cholesterol-rich diet supplemented with grape pomace, a significant reduction in serum total cholesterol, LDL-cholesterol, and an atherogenic lipid index was reported [42]. This effect was also detected in nonsmoking patients receiving grape-derived antioxidant dietary fiber [7]. Similarly, consumption of green tea beverages or extracts was also associated with significant total cholesterol and LDL-cholesterol lowering in humans [43]. However, these previous findings diverge from those seen in our model, in which the intake of RWGP-supplemented diet did not reduce total or lipoprotein cholesterol levels in atherogenic diet-fed SR-B1 KO/ApoER61^h/h^ mice. The lack of effect of RWGP on lipoprotein cholesterol levels found in our study may be explained by differences in species, dietary antioxidant and fiber composition, and dyslipidemic pattern compared with previous studies.

Interestingly, RWGP supplementation modulated the systemic inflammatory status induced by the intake of an atherogenic diet as shown by reduction in TNF-α circulating levels. TNF-α is a pro-inflammatory cytokine also considered as a pro-atherogenic factor [44] and high levels have been found in patients with coronary atherosclerosis [45]. Malvidin [46] and catechin [47], among other polyphenolic antioxidants, inhibit the production of TNF-α and other downstream pro-inflammatory cytokines. Thus, the reduction in circulating TNF-α levels seen in our work may be related to the anti-inflammatory properties of polyphenols and anthocyanins. In fact, grape pomace prevented the pro-inflammatory response of microglia cells in response to lipopolysaccharide (LPS), decreasing the expression of several inflammatory molecules, including TNF-α and IL-1β [48].

Unexpectedly, HFC-Control-fed SR-B1 KO/ApoER61^h/h^ mice also presented high levels of circulating IL-10, an anti-inflammatory cytokine, which were attenuated by RWGP supplementation. This apparent paradoxical response in IL-10 versus TNF-α has been described in other inflammatory diseases, and probably represents the need for balance between pro-inflammatory and anti-inflammatory signals that are required to maintain immune homeostasis. For instance, children with cystic fibrosis show increased TNF-α levels counterbalanced by simultaneous synthesis of IL-10 [49].

SR-B1 KO/ApoER61^h/h^ mice rapidly respond to the challenge of an atherogenic diet with plaque formation both in the aortic root and coronary arteries, recapitulating many features of human ASCVD [20]. SR-B1 KO/ApoER61^h/h^ mice fed with HFC-Control diet readily developed plaques in highly vulnerable regions along the arterial tree, in agreement with previously published data [12,28,29,50]. Supplementation with RWGP significantly reduced atherosclerotic lesion development. Similar results have been described in SR-B1/ApoE dKO mice that received pomegranate extract in drinking water—also known to be rich in polyphenolic compounds [12]—and LDL receptor KO mice supplemented with naringenin [51]. In addition, our studies using vitamin C and E in atherogenic diet-fed SR-B1 KO/ApoER61^h/h^ mice showed a reduction in atherosclerosis development with improvement in HDL composition and function [29]. Our results also disclose a significant reduction in the vascular uptake of the MRI contrast agent gadofosveset in the group receiving RWGP, suggesting a reduction in endothelial permeability. A decrease in contrast agent uptake has also been reported in mouse models of atherosclerosis treated with statins and anti-inflammatory therapies [52]. 

Additionally, attenuated atherosclerosis development was associated with reduced percentage of hearts exhibiting myocardial infarction and improvement in cardiac ejection fraction in the HFC-RWGP group. Recovery of heart function has also been reported in SR-B1 KO/ApoER61^h/h^ mice after the administration of the immunosuppressant FTY720 [50,53]. Our findings are interesting since the compositional analysis of HFC-RWGP diet showed an increase in total tocopherol content, more specifically in α-tocopherol, the most active member of vitamin E lipid-soluble antioxidant compounds. A meta-analysis indicated that vitamin E supplementation without other antioxidants significantly reduced myocardial infarction risk by 18% [54]. This clinical effect was essentially driven by a reduction of fatal myocardial infarction while no effect was observed for nonfatal ischemic heart disease, suggesting a protective role of vitamin E supplementation alone whereas the use of a combination of antioxidants as anti-atherosclerotic therapy is ineffective and potentially harmful [54]. A recent study has shown potential benefits of acute treatment with vitamin E in a mouse model of cardiac ischemia/reperfusion, reducing infarct size and oxidative stress, and preserving cardiac function [55]. In addition, supplementation of tocotrienol and tocopherol combination decreased infarct volumes and inflammatory markers in ischemic brains of mice [56].

Management of residual risk is one of the major issues in treatment of patients after an ischemic event and controlling remnant inflammatory responses has been an important focus of several clinical trials seeking adjuvant therapies to statin treatment. In this context, diet remains as a key factor to be modified. Both cohort studies and intervention trials have shown an association between adherence to the Mediterranean diet and lower cardiovascular risk [6]. Based on the available literature and our results, incorporating functional food components into patients’ diets may be beneficial in the management of residual risk. Indeed, we have reported that daily consumption of RWGP by patients with at least one component of metabolic syndrome, along with their regular diet, significantly improved blood pressure, glycaemia, and postprandial insulin levels together with an overall improvement of antioxidant defense [18]. Similarly, intake of beef burgers prepared with RWGP showed better fasting glucose, reduced insulin resistance, and improved plasma antioxidant levels and oxidative damage markers in male volunteers with metabolic syndrome [57]. In addition, intake of a drink prepared from red grape pomace can deliver significant amounts of different phenolic metabolites in humans improving insulin sensibility [58,59]. Taken together, these results strongly suggest that supplementing RWGP into food intake of either CVD patients or individuals at risk may be a co-adjuvant not only in preventing cardiovascular events, but also attenuating development and progression of comorbidities known to be part of the metabolic syndrome.

In summary, this study showed that the RWGP-supplementation significantly attenuated atherosclerosis development in SR-B1 KO/ApoER61^h/h^ mice in association with increased overall plasma antioxidant activity and modulation of inflammation. We also substantiated the value of non-invasive MRI in this model, emphasizing the importance of SR-B1 KO/ApoER61^h/h^ as a valuable tool to explore potential therapies that may help in primary and secondary prevention of ischemic ASCVD events. However, more studies are needed to establish if these RWGP-associated positive outcomes in mice can be translated into the human context. However, these results—in addition to our previous published data [18,19,57]—are encouraging on the potential benefits of incorporating wine grape pomace into a regular diet in order to improve cardiovascular health and decrease ASCVD.

## Figures and Tables

**Table 1:** Nutritional composition of atherogenic diets supplemented with 20% chow (high fat, high cholesterol, and cholic acid (HFC)-Control), 20% red wine grape pomace (RWGP) flour (HFC-RWGP), or 10% chow/10% oat fiber (HFC-Fiber).

	HFC-Control	HFC-RWGP	HFC-Fiber
**Proximate analysis and fiber** (g/100 g of supplemented diet)			
Fat	14.5	15.1	13.4
Protein	20.1	17.2	17.2
Carbohydrates ^a^	37.4	30.6	33.2
Dietary fiber	15.0	22.1	22.8
Soluble	2.6	3.6	2.5
Insoluble	12.4	18.5	20.3
Ash	5.1	5.1	4.4
Moisture	8.0	9.9	9.1
**Antioxidants and antioxidant capacity**			
Polyphenols (mg GE/g)	1.9 ± 0.08	6.3 ± 0.42	1.6 ± 0.10
α-tocopherol (μg/g)	2.1 ± 0.13	8.2 ± 0.88	1.7 ± 0.08
γ-tocopherol (μg/g)	15.2 ± 1.20	18.3 ± 1.16	9.2 ± 0.41
δ-tocopherol (μg/g)	7.9 ± 0.62	5.2 ± 0.74	3.9 ± 0.21
Total tocopherols (μg/g)	25.2 ± 1.95	31.7 ± 2.78	14.8 ± 0.70
Vitamin C (μg/g)	200.8 ± 8.58	248.6 ± 8.12	260.1 ± 3.27
ORAC (μmoles TE/g)	35.8 ± 2.61	75.4 ± 3.61	36.3 ± 2.05

Values are mean ± SD. GE: gallic equivalent; ORAC: oxygen radical absorbance capacity; TE: trolox equivalent. Values represent averages from two to three independent measurements. ^a^ Nitrogen-free extract minus dietary fiber.

**Table 2:** RWGP supplementation reduces macroscopic myocardial infarction in atherogenic diet-fed SR-B1 KO/ApoER61^h/h^ mice.

	Day 7; Number of Hearts (%)			Day 14; Number of Hearts (%)		
Treatment	Infarcted	Non-Infarcted	Total	Infarcted	Non-Infarcted	Total
HFC-Control	1 (14.3%)	6 (85.7%)	7	7 (100%)	0 (0%)	7
HFC-RWGP	0 (0%)	7 (100%)	7	1 (14.3%)	6 (85.7%) ^a^	7

^a^*p* < 0.0001 vs. HFC-Control; Chi-squared test.

## Supplementary Materials

The following are available online at https://www.mdpi.com/2072-6643/11/9/2135/s1, Table S1: Nutritional composition of RWGP flour and isolated oat fiber 780. Figure S1: Effect of RWGP supplementation on body weight of atherogenic diet-fed SR-B1 KO/ApoER61^h/h^ mice. Figure S2: Effect of RWGP supplementation on spleen and liver weights of atherogenic diet-fed SR-B1 KO/ApoER61^h/h^ mice. Figure S3: Effect of RWGP supplementation on plasma liver enzymes in atherogenic diet-fed SR-B1 KO/ApoER61^h/h^ mice. Figure S4: Aortic arch of atherogenic diet-fed SR-B1 KO/ApoER61^h/h^ mice reconstructed from bright-blood MR acquisition.
