# Supplementary material for: Red Wine Grape Pomace Attenuates Atherosclerosis and Myocardial Damage and Increases Survival in Association with Improved Plasma Antioxidant Activity in a Murine Model of Lethal Ischemic Heart Disease
Source: Nutrients. 2019 Sep 6;11(9):2135. doi: 10.3390/nu11092135 (PMC6770693; doi:10.3390/nu11092135)
Supplement: Supplementary file 1 [file nutrients-11-02135-s001.pdf]

Supplemental Material

TABLE S1: Nutritional composition of RWGP flour and isolated Oat Fiber 780.

	**RWGP** (g/100g)	**Oat Fiber 780** (g/100g)
**Proximate analysis and fiber content**		
Fat	7.8	0.04
Protein	11.7	0.0
Carbohydrates[a]	17.0	92.8
Dietary fiber	47.7	92.8[b]
Soluble	3.5	0.4
Insoluble	44.2	92.4
Ash	8.4	2.6
Moisture	7.5	7.0

Values are mean ± SD

[a] Nitrogen-free extract minus dietary fiber

[b] AOAC 991.43

[*] The values represent averages of two to three independent measurements.

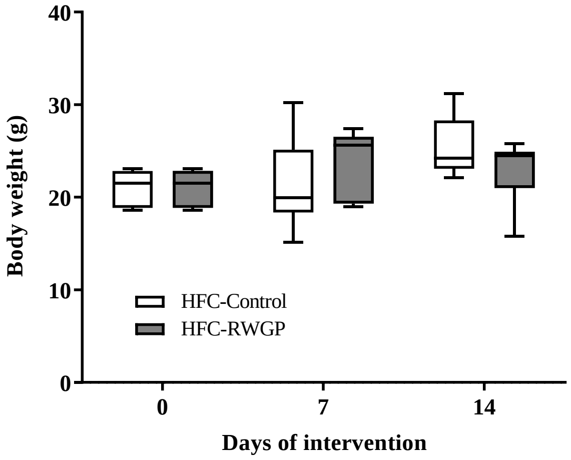

FIGURE S1: Effect of RWGP supplementation on total body weights of of SR-B1 KO/*Apoe*R61$^{h/h}$ mice fed with atherogenic diet. Data are shown in box plots (box, 25th to 75th percentiles; whiskers, median and IQR) (n = 5 to 7) based on two-way ANOVA.

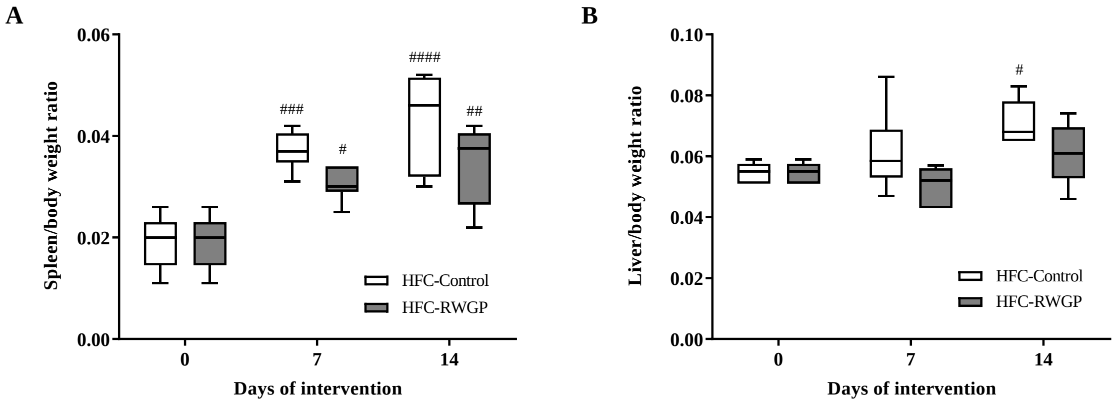

FIGURE S2. Effect of RWGP supplementation on spleen and liver weights of SR-B1 KO/*Apoe*R61$^{h/h}$ mice fed with atherogenic diet. (A) Spleen and (B) liver weights, relative to body weight, at baseline and after 7 and 14 days of HFC-supplemented diet. Data are shown in box plots (box, 25th to 75th percentiles; whiskers, median and IQR) (n = 5 to 7). $^{\#}$ P < 0.01 and $^{\#\#}$ P < 0.01 compared to day 0; * P < 0.01 vs HFC-Control based on two-way ANOVA.

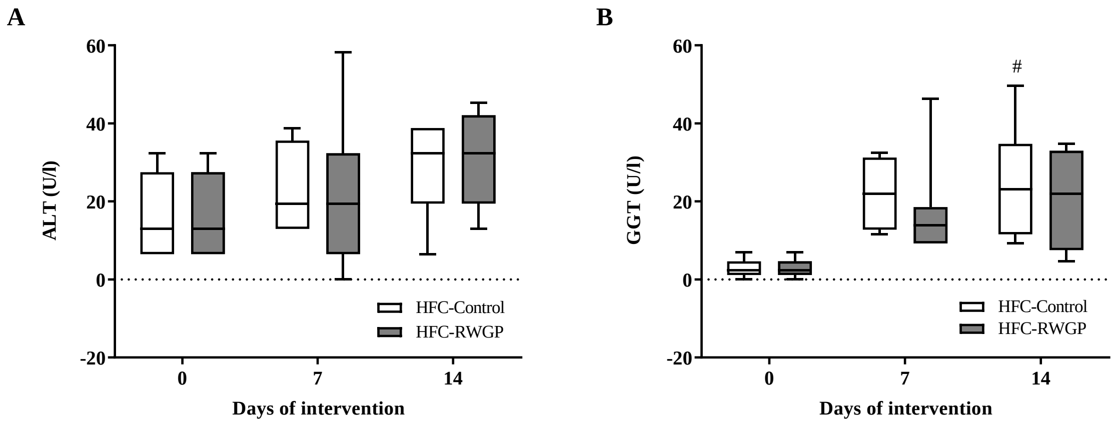

FIGURE S3. Effect of RWGP supplementation on plasma liver enzymes. Plasma levels of (A) alanine transaminase (ALT) and (B) gamma-glutamyltransferase (GGT) at baseline and after 7 and 14 days of HFC-supplemented diet. Data are shown in box plots (box, 25th to 75th percentiles; whiskers, median and IQR) (n = 5 to 7). # $P < 0.05$ compared to day 0 based on two-way ANOVA.

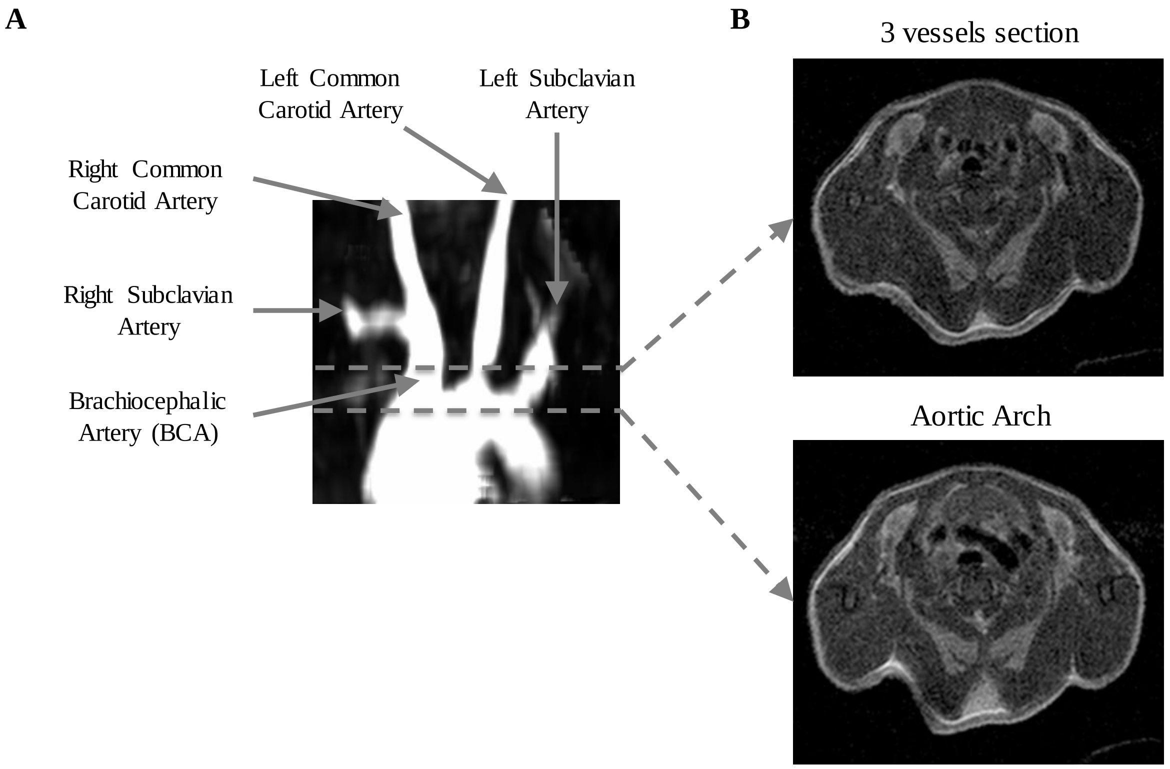

FIGURE S4. Reconstruction of aortic arch/supra-aortic vessels in mice subjected to bright-blood MRI. (A) Aortic arch of mice reconstructed from bright-blood MR acquisition. Supra-aortic vessels are shown: brachiocephalic artery, subclavians arteries, and carotid arteries. (B) Axial section of a black-blood acquisition at the level of the aortic arch and the 3 supra-aortic vessels.
